# In Silico Identification of Cassava Genome-Encoded MicroRNAs with Predicted Potential for Targeting the ICMV-Kerala Begomoviral Pathogen of Cassava

**DOI:** 10.3390/v15020486

**Published:** 2023-02-09

**Authors:** Muhammad Aleem Ashraf, Babar Ali, Judith K. Brown, Imran Shahid, Naitong Yu

**Affiliations:** 1 Institute of Tropical Biosciences and Biotechnology, Chinese Academy of Tropical Agricultural Sciences, Haikou 571101, China; 2 Institute of Biological Sciences, Faculty of Natural and Applied Sciences, Khwaja Fareed University of Engineering and Information Technology, Rahim Yar Khan 64200, Pakistan; 3 School of Plant Sciences, The University of Arizona, Tucson, AZ 85721, USA; 4 Department of Pharmacology and Toxicology, Faculty of Medicine, Umm Al-Qura University, Makkah 21955, Saudi Arabia

**Keywords:** *begomovirus*, computational miRNA algorithms, Indian cassava mosaic virus-Kerala, non-coding RNA, RNA interference, R-language, virus resistance

## Abstract

Cassava mosaic disease (CMD) is caused by several divergent species belonging to the genus *Begomovirus* (*Geminiviridae*) transmitted by the whitefly *Bemisia tabaci* cryptic species group. In India and other parts of Asia, the Indian cassava mosaic virus-Kerala (ICMV-Ker) is an emergent begomovirus of cassava causing damage that results in reduced yield loss and tuber quality. Double-stranded RNA-mediated interference (RNAi) is an evolutionary conserved mechanism in eukaryotes and highly effective, innate defense system to inhibit plant viral replication and/or translation. The objective of this study was to identify and characterize cassava genome-encoded microRNAs (mes-miRNA) that are predicted to target ICMV-Ker ssDNA-encoded mRNAs, based on four in silico algorithms: miRanda, RNA22, Tapirhybrid, and psRNA. The goal is to deploy the predicted miRNAs to trigger RNAi and develop cassava plants with resistance to ICMV-Ker. Experimentally validated mature cassava miRNA sequences (*n* = 175) were downloaded from the miRBase biological database and aligned with the ICMV-Ker genome. The miRNAs were evaluated for base-pairing with the cassava miRNA seed regions and to complementary binding sites within target viral mRNAs. Among the 175 locus-derived mes-miRNAs evaluated, one cassava miRNA homolog, mes-miR1446a, was identified to have a predicted miRNA target binding site, at position 2053 of the ICMV-Ker genome. To predict whether the cassava miRNA might bind predicted ICMV-Ker mRNA target(s) that could disrupt viral infection of cassava plants, a cassava locus-derived miRNA–mRNA regulatory network was constructed using Circos software. The in silico-predicted cassava locus-derived mes-miRNA-mRNA network corroborated interactions between cassava mature miRNAs and the ICMV-Ker genome that warrant in vivo analysis, which could lead to the development of ICMV-Ker resistant cassava plants.

## 1. Introduction

Cassava *Manihot esculenta* Crantz is a tropical food crop of societal and agricultural importance in Africa, the tropical Americas, and Asia [1]. Cassava production in the mainland of Southeast Asia continues to increase in acreage. The cassava genome (2*n* = 36) [2] is heterozygous; several hundred genomes of cultivars and wild accessions have been re-sequenced [3,4]. Cassava mosaic disease (CMD) is caused by at least ten species in the genus *Begomovirus* (*Geminiviridae)* that are endemic to Africa and Asia [5,6]. Begomoviruses are transmitted by the whitefly *Bemisia tabaci* (Gennadius) (Aleyrodidae: Hemiptera) cryptic species complex [7,8,9,10,11]. Cassava cultivars grown in Asia lack tolerance or resistance to cassava mosaic begomovirus infection [5,12,13]. Extensive yield losses have been attributed to CMD infection, ranging from 20 to 90%, reported worldwide [14]. The Indian cassava mosaic virus-Kerala (ICMV-Ker) bipartite single-stranded (ss) DNA-A is 2743 nucleotides in length [15,16,17]. The ICMV-Ker genome encodes six proteins that are transcribed bidirectionally from the large intergenic region (LIR). The virion-sense and complementary-sense strands encode ORFs AV1 and AV2, and ORFS AC1, AC3 and AC4, respectively [18,19].

MicroRNAs (miRNAs) represent a class of endogenous, small, non-coding single-stranded (ss) RNA regulatory molecule of 20–24 nucleotides (nt). The long single-standard primary miRNAs (pri-miRNAs) are produced by nuclear-encoded *MIR* genes, and govern gene regulation and cell growth, regulate host–virus interactions, and control gene expression at the post-transcriptional level [20,21]. The biogenesis of miRNAs is a complex multistep process entailing transcription, processing, and nuclear export, and also involves the synthesis of pri-miRNA, processing into stem-loop structures, development of intermediate duplexes (miRNA/miRNA*), miRNA stabilization by 2′-o-methylation, incorporation into RNA-induced silencing complexes (RISCs), and miRNA degradation. The complementary target mRNA is degraded by RISC [22,23,24,25]. MicroRNA-mediated RNA interference (RNAi) is an innate defense mechanism involving gene regulation in response to host–virus interactions, and therefore is an inherent mechanism for enhancing or inhibiting virus infection at the post-transcriptional level [26,27,28].

Artificial microRNA (amiRNA) technology is one of the most effective antiviral defense strategies to protect crop plants against infection by plant viruses. RNAi-mediated gene silencing through amiRNA has been used to develop plants with resistance to diverse plant viral species, among which are cucumber green mottle mosaic virus [29,30], rice stripe virus [31], turnip mosaic virus [32,33], plum pox virus [34], cucumber mosaic virus [35], potato virus Y (PVY) [36], cotton leaf curl Kokhran virus-Burewala [37], cymbidium mosaic virus, and odontoglossum ringspot virus [38].

The identification, analysis, and experimental validation of mature miRNAs in cassava is an important initial step in evaluating host–virus interactions, which are aided by the use of regulatory, network analysis of the host plant, in the context of environmental stresses, including virus infection [39,40,41,42]. Recently, 175 conserved mature cassava locus-derived mes-miRNAs were experimentally validated and made available in the miRBase database [43]. The mature cassava miRNAs have been associated with immunity against biotic and abiotic stress [42,44,45], of which a subset of high-confidence, mature-miRNAs are hypothesized to target the ICMV-Ker genome.

In this study, an integrative computational approach for combatting ICMV-Ker infection was used to predict the presence of target-binding sites of cassava locus-derived mes-miRNAs in the ICMV-Ker genome. The goal is to identify homologous amiRNAs that for expression in transgenic cassava plants to confer resistance to ICMV-Ker. The predicted cassava locus-derived mes-miRNAs were further analyzed for the predicted involvement in cassava host plant–ICMV-Ker begomoviral interactions to identify virus-specific targets of interest. The goal is to express the most promising in silico cassava locus-derived mes-miRNAs in transgenic cassava plants to achieve amiRNA-mediated resistance to the recently emergent, bipartite ICMV-Ker begomovirus in cassava crops in Southeast Asia.

## 2. Materials and Methods

### 2.1. Cassava Biological Data Retrieval

In a previous study, 175 mature cassava microRNAs (*Manihot esculenta*-microRNA) (mes-miRNA156-mes-miR11892) have been identified (Supplementary Table S1 (Accession IDs: MIMAT0029165-MIMAT0045988). The latter mature cassava miRNAs were accessed from the miRNA registry (miRBase, version 22) (http://mirbase.org/ accessed on 26 May 2019) biological database. The miRBase is the central primary public online repository for miRNAs with a user-friendly web interface to access published miRNA sequences and annotations. The full-length genome (2743 bases) of ICMV-Ker (IN-Ker2-021) (Accession number AJ575819) was downloaded from the National Center for Biotechnology Information NCBI GenBank database (http://ncbi.nlm.nih.gov) (accessed on 26 May 2019) [46].

### 2.2. Potential miRNA Target Prediction in ICMV-Ker Genome

The in silico prediction of miRNA-mRNA target sites is an initial step towards discovering gene regulatory networks controlled by miRNA. Several computational algorithms are available for predicting potential miRNA target sites in viral mRNA conducive to effective silencing. Each computational algorithmic tool set specific criteria for miRNA prediction. An integrative bioinformatic approach involves the combined use of publicly available in silico algorithms. Among the most widely used are miRanda, RNA22, Tapirhybrid, and psRNATarget. The latter four algorithms were used to screen the ICMV-Ker genome and predict the ‘most effective’ miRNA target sites of the cassava miRNAs (Table 1). The cassava locus-derived mes-miRNA sequences and the ICMV-Ker genome-predicted transcripts (in FASTA format) were analyzed using the default parameters.

### 2.3. miRanda

The miRanda is a seed-based scanning computational algorithm, and has been used as a standard for miRNA-mRNA target prediction, following its releases in 2003 [47]. It considers features predictive of RNA duplex dimerization, target site location, and sequence complementarity. Minimum free energy (MFE) and cross-species target conservation are characteristics of the miRNA algorithm that distinguish it from other algorithms [48]. The miRanda algorithm (C programming language) was downloaded from the online source website (http://www.microrna.org/) (accessed on 9 June 2019). Target prediction was carried out using the default parameters (MFE threshold: −20 Kcal/mol, score threshold: 140.00, gap open penalty: −9∙000, gap extend penalty: −4∙000 and scaling parameter: 4∙00). The MFE statistical parameter is used to evaluate miRNA-target pair strength.

### 2.4. RNA22

The RNA22 algorithm is a diverse, web-based algorithm that uses a pattern-recognition based approach, available online http://cm.jefferson.edu/rna22v1.0/ (accessed on 20 July 2019) [49]. It predicts statically significant target patterns using minimum folding energy (MFE), site complementarity, and non-seed-based interaction [50]. Default parameters selected to identify miRNA target binding sites of cassava mature miRNAs in the ICMV-Ker genome were output format (heteroduplexes); sensitivity (63%), specificity (61%), and maximum folding energy for heteroduplex was set as −15.00 Kcal/mol.

### 2.5. Tapirhybrid

The Tapirhybrid algorithm is a web-based, rapid, and precise plant miRNA target prediction algorithm [51] used to deliver precise miRNA target predictions, including target mimics, with FASTA and RNAhybrid search options, it has been developed for seed and sequence-based predictions and identifies miRNA-target interactions, and is available online: http://bioinformatics.psb.ugent.be/webtools/tapir (accessed on 19 July 2020). The standard default settings—score <9 and MFE ratio <0.2—were used for this study.

### 2.6. psRNATarget

The psRNATarget algorithm is a highly sensitive, web-based plant miRNA prediction algorithm [52,53] for identifying target binding sites of plant miRNAs, based on complementary scoring schema. The algorithm predicts the inhibition pattern of cleavage for plant miRNAs. The FASTA sequence of the ICMV-Ker genome and 153 published cassava miRNAs were preloaded at the psRNATarget web server (available online: http://plantgrn.noble.org/psRNATarget) (accessed on 26 May 2019). The miRNA–mRNA target binding sites were predicted using the default criteria: an expectation cut-off value of 7.0 and the mode of inhibition set to ‘cleavage’ (File S1).

### 2.7. Mapping of Network-Based miRNA-Target Interactions

A Circos plot was drawn using CIRCOS algorithm and package v0.69-9 [54].

### 2.8. RNAfold

RNAfold is a recently available web-based algorithm that predicts secondary structures from a single-stranded (ss) miRNA precursor. Precursor sequences of cassava consensus miRNAs in FASTA format were preloaded in the RNAfold web server (available online: http://rna.tbi.univie.ac.at/cgi-bin/RNAWebSuite/RNAfold.cgi) (accessed on 6 September 2022), and analyzed with the user-defined default settings [55].

### 2.9. Free Energy (ΔG) Estimation of Duplex Binding

RNAcofold is a newly developed algorithm for estimating co-folding free energy (ΔG) of RNA duplex sequences, based on minimum free energy and base-pairing patterns of miRNA-mRNA target duplex molecules. It is specifically implemented for evaluating the mRNA and miRNA duplex interactions. FASTA sequences of the duplex pair from miRanda analysis were preloaded in the RNAcofold web server (available online: http://rna.tbi.univie.ac.at/cgi-bin/RNAWebSuite/RNAcofold.cgi) (accessed on 20 November 2022) [56].

### 2.10. Statistical Analysis

All of the computational biological data sets resulting from the miRNA target predictions were summarized as graphical representations using R-language (version 3.1.1, software version 3.5.1) [57].

## 3. Results

### 3.1. Cassava Locus-Derived mes-miRNAs Targeting ICMV-Ker Genome

**Figure 1:**
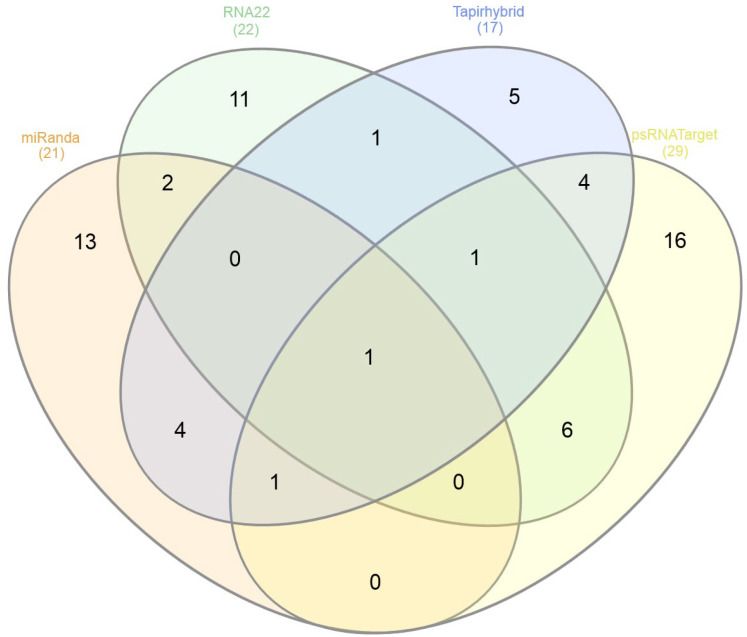
Venn diagram of cassava locus-derived mes-miRNA-target pairs predicted to bind the ICMV-Ker genome. The miRanda, RNA22, Tapirhybrid, and psRNATarget databases were mined to predict potential binding sites of cassava locus-derived mes-miRNAs. The degree of overlap between computational tools was observed at potential target-binding site level. The intersection of four tools’ graph showed a single common miRNA: mes-miR1446a.

The in silico predictions of cassava locus-derived mes-miRNAs with the potential to target viral ORFs encoded by the ICMV-Ker genome from among the 175 cassava locus-derived mature mes-miRNAs in the ICMV-Ker single-stranded (ss) DNA genome revealed mes-miRNAs derived from MIR genes at specific cassava genomic loci. The predicted ICMV-Ker genome sequences targeted by cassava locus-derived mes-miRNAs based on the miRanda algorithm predicted 21 miRNA-mRNA target pairs, RNA22: 22 cassava miRNAs and 22 loci. The Tapirhybrid algorithm identified 17 cassava genome locus-derived mes-miRNA-target pairs. In contrast, the psRNATarget algorithm predicted cleavable targets for 29 cassava locus-derived mes-miRNAs targeting at 33 locus positions in the ICMV-Ker genome (Figure 1) (File S2).

### 3.2. Cassava miRNAs Targeting Virion-Sense ORFs

The begomoviral AV1 ORF (295-1065) (770 bases) encodes the coat protein (CP), which is essential for encapsidation of the viral ssDNA genome into virions transmitted by the whitefly vector, and for cell-to-cell movement [58,59,60]. Certain mutations in the coat protein may result in altered competency or rate of transmission by different cryptic species or mitotypes of the whitefly vector [61].

**Figure 2:**
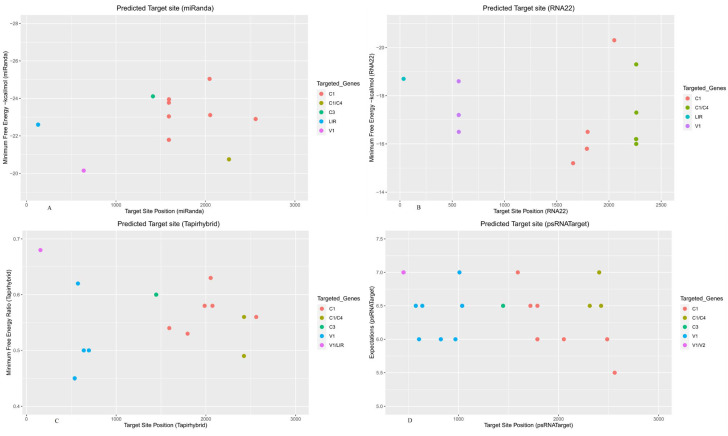
Predicted target binding sites of cassava mes-miRNAs in the ICMV-Ker genome were identified using four selected miRNA-target prediction algorithms. (**A**) The miRNA target binding sites predicted by miRanda. (**B**) The miRNA targets obtained by RNA22. (**C**) Tapirhybrid-predicted potential target binding sites. (**D**) Predicted miRNA targets based on the psRNATarget algorithm. Each colored dot represents one miRNA target binding site, within specific ICMV-Ker genome coding regions. Each viral gene is indicated by a different color, as indicated by the legend shown to the right of the graph, respectively.

The miRanda algorithm predicted binding of one miRNA: mes-miR2950 at nucleotide position 639 (Figure 2A and Table 2). Further, four miRNAs were predicted to target begomoviral AV1: mes-miR535 (a, b, c and d) at nucleotide position 563, as indicated by RNA22 (Figure 2B and Table 2). Additionally, the Tapirhybrid algorithm predicted five cassava locus-derived mes-miRNAs: mes-miR160e (locus 574), mes-miR395e (locus 156), mes-miR482e (locus 538), mes-miR535c (locus 696), and mes-miR2950 (Figure 2C and Table 2). The V1 ORF was targeted by sixteen predicted miRNAs: mes-miR160 (e, f) (locus 574), mes-miR171 (g, h, i, j, and k) (locus 1037), mes-miR397 (loci 970, 1010), mes-miR2111 (a, b) (locus 824), and mes-miR2950 (locus 639), based on the psRNATarget algorithm. Further overlapping segments of the AV1 and AV2 ORFS were predicted targets of the mes-miR394 family, at locus 452, according to the psRNATarget algorithm (Figure 2D and Table 2) (Supplementary Table S2).

### 3.3. Cassava miRNAs Targeting Complementary-Sense ORFs

The AC1 ORF (1552-2610 nt) complementary strand encodes a replication-associated protein, whereas the AC3 ORF (1062-1466 nt) encodes a replication enhancer protein. These viral proteins are essential for functions, including viral DNA replication, transcription, transmission, cell-to-cell movement, suppression, and gene regulation [62,63,64,65,66]. Several predicted binding sites were identified for the AC1 gene of ICMV-Ker based on the miRanda algorithm and were mes-miR399e located in the locus 2561, mes-miR477 (a, b, c, d, e, f, g, h, I, and k) in locus 2592, and mes-miR1446 (a, b) in locus 2053. Based on miRanda analysis, the cassava locus-derived mes-miR390b was predicted to target the C3 coding region from locus 1413. This algorithm also predicted two miRNAs: mes-miR59 (c, d) at the common locus, 2263, which targeted an overlapping segment of the AC1 and AC4 genes, respectively (Figure 2A and Table 2). RNA22 identified binding sites of the cassava miRNAs: mes-miR395 (a, b, c, and d) from the common nucleotide locus position 1789, mes-miR395e from locus position 1657, mes-miR1466a from locus position 2051, and mes-miR2118 from locus 1796. Finally, predicted miRNAs targeting overlapping region of AC1 and AC4 genes were: mes-miR159 (a-3p, b, c, and d) from the common locus position 2261 and mes-miR319 (a, b, e, f, g, and h) from the common locus position 2262, by RNA22 (Figure 2B and Table 2).

The Tapirhybrid algorithm identified ten predicted miRNAs: mes-miR172 (e, f) from the common locus 1986, mes-miR399e at locus 2561, mes-miR477 (f, g) from a common locus 1592, mes-miR477h from locus 1796, mes-miR482d from locus 2073, and mes-miR1446a from locus 2053. Additionally, mes-miR530 (a, b) targeted the overlapping segments of AC1 and AC4 from the common locus 2425 (Figure 2C and Table 2).

The psRNATarget algorithm analysis predicted that the ICMV-Ker ACI ORF was targeted by fifteen miRNAs: mes-miR159 (a, b) from the common locus 1719, mes-miR395 (a, b, c, and d) from the common nucleotide locus position 1789, mes-miR399b from locus 2561, mes-miR827 from locus 2486, mes-miR1446a from locus 2053, and mes-miR2275 from locus 1593 (Figure 2C and Table 2). Further, psRNATarget identified predicted miRNAs generated from the following loci in cassava that targeted an overlapping region of ICMV-Ker AC1 and AC4 genes, mes-miR159 (a, b) at the common locus 2406, mes-miR397 at locus 2312, and mes-miR530a at locus 2425 (Figure 2D and Table 2).

### 3.4. Cassava miRNAs Targeting Large Intergenic Region

The DNA-A genome of begomoviruses possesses a LIR that functions as a bidirectional promoter, essential for replication and expression of the viral genes, AC1 (Rep) and AV1 (CP) [67,68]. The miRanda algorithm predicted hybridization of mes-miR393 (a, b, c, and d) from the common locus 130 in the LIR of the ICMV-Ker genome (Figure 2A and Table 2). Predicted candidate miRNAs from cassava (mes-miR399h) were identified that targeted the ICMV-Ker LIR at nucleotide positions 35–54, based on the RNA22 algorithm (Figure 2B and Table 2). The Tapirhybrid algorithm predicted the target binding site, mes-miR395e from locus 156 that targeted the overlapping AV1 and LIR region (Figure 2C and Table 2). No target binding sites were predicted within the LIR, by the psRNATarget algorithm (Figure 2D and Table 2).

### 3.5. Identification of Common and Unique Cassava miRNAs

**Figure 3:**
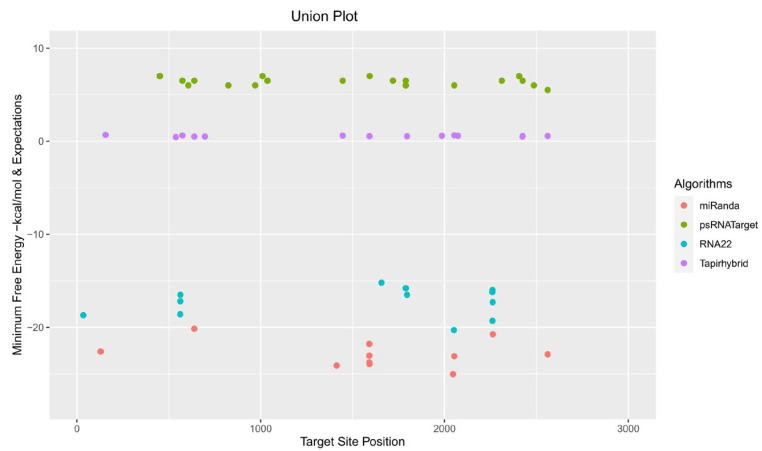
Union plot showing cassava miRNA-target pairs, predicted by all four algorithms used to analyze the cassava host-begomoviral data sets in this study.

Among the predicted cassava locus-derived candidate mes-miRNAs, seventeen miRNAs (mes-miR159 (a, b, c, and d), mes-miR160e, mes-miR395 (a, b, c, and d), mes-miR399e, mes-miR403 (a, b), mes-miR477 (f, g, h), mes-miR530a, and mes-miR535c) were predicted by at least two of the algorithms. Of seventeen consensus cassava locus-derived mes-miRNAs, one miRNA (mes-miR2950 at locus 639) was detected by three algorithms (miRanda, Tapirhybrid and psRNATarget). The mes-miR1446a was predicted by all four algorithms, making it the only unique cassava miRNA identified in this study (Figure 1 and Figure 3) (Table 2 and Table 3) (Tables S2 and S3 and File S2).

### 3.6. Predicting Consensual Cassava miRNAs and Silencing ICMV-Ker Genome Sequences

**Figure 4:**
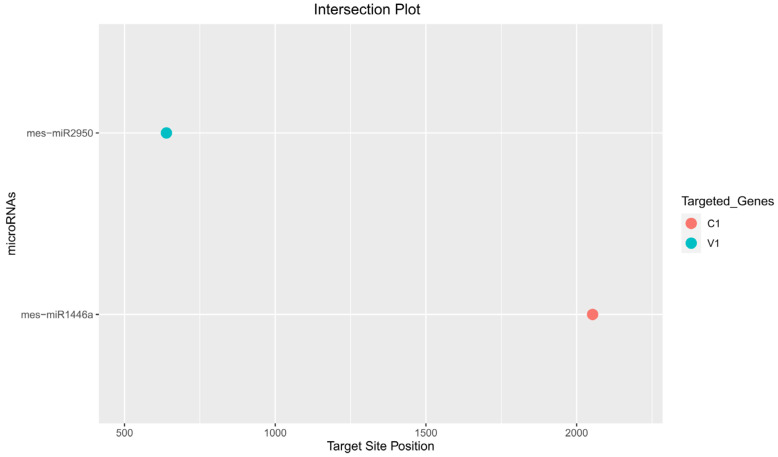
Intersection plot representing consensus cassava miRNAs predicted by at least three algorithms at common genomic loci targeting specific ICMV-Ker genes. The red dot represents cassava miRNA (mes-miR1446a) targeting C1 gene of ICMV-Ker, which was predicted by the four algorithms in this study.

Of the 175 targeting mature cassava miRNAs, 15 cassava miRNAs (mes-miR159 (c, d) (locus 2261), mes-miR160e (locus 574), mes-miR395 (a, b, c, and d) (locus 1789), mes-miR399e (locus 2561), mes-miR403 (a, b) (locus 1446), mes-miR477 (f, g) (locus 1592), and mes-miR530a (locus 2425) were identified by consensus between two algorithms (Table 3 and Table 4). Of the 15 consensus miRNAs, one cassava miRNA, mes-miR2950 at locus 639, with an MFE of −20.15 Kcal/mole, was predicted at the consensus locus by at least three of the algorithms (Table 3 and Figure 4).

In this study, based on all four algorithms, mes-miR1466a was the top predicted ‘effective’ cassava locus-derived candidate mes-miRNA, and harbored potential target binding sites at the consensus nucleotide position 2053 (Figure 4). The miRanda and RNA22-MFE values for the cassava locus-derived mes-miRNA-target pair were −23.11 and −20.3 Kcal/mol, respectively. Finally, the mes-miR1466a was predicted to bind to the ICMV-Ker AC1 gene (Table 3, Table 4 and Table 5).

### 3.7. Association of Cassava miRNAs with Corresponding Gene-Targets

**Figure 5:**
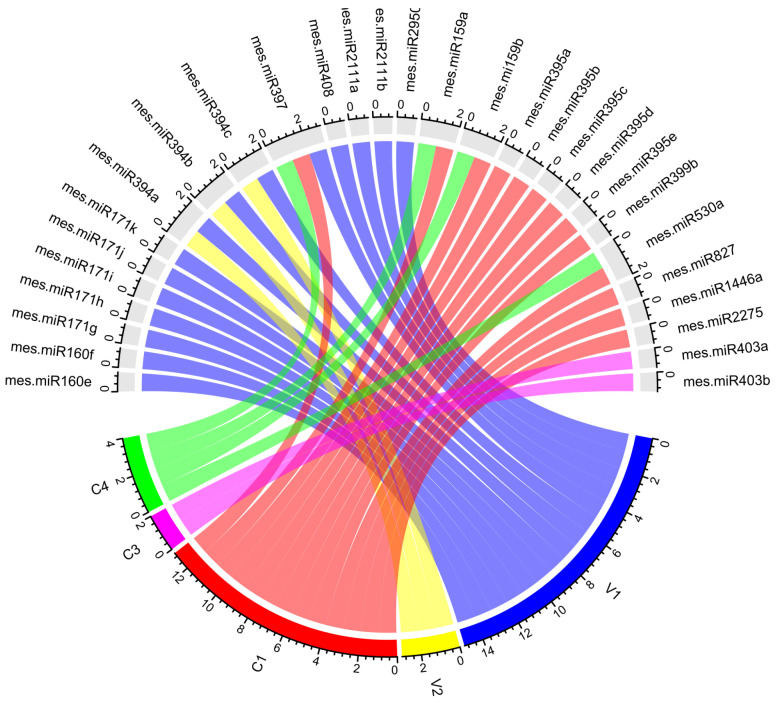
Integrated Circos plot show cassava locus-derived mes-miRNA-mRNA target interactions. The colored connection lines represent ICMV-Ker genomic sequences. The plots indicate the target predictions and interactions between the cassava locus-derived mes-miRNAs and viral genes.

A comprehensive, global purview of the predicted host–virus interactions was visualized by constructing a Circos plot (“Circos” software) that integrated the biological data, comprising cassava locus-derived mes-miRNAs and their predicted ICMV-Ker genomic target genes (ORFs). For cassava miRNAs with predicted cassava host plant-viral gene-target interactions, the plot shows the cassava miRNAs and their ICMV-Ker targets by psRNATarget analysis (Figure 5) (File S3).

### 3.8. Prediction of Consensual Secondary Structures

Computational predictions of the consensus cassava locus-derived mes-miRNAs (mes-miR1446a and mes-miR2950) were corroborated by secondary structure predictions. The pre-miRNA sequences were manually curated. Characteristic features of precursor miRNAs were evaluated for stability of the respective secondary structure (Table 6).

### 3.9. Evaluation of Free Energy (ΔG) of mRNA-miRNA Interactions

The predicted cassava locus-derived mes-miRNA-mRNA target pairs were evaluated and validated by calculating the free energies (Δ*G*) of duplexes (Table 7).

## 4. Discussion

The ICMV-Ker is a bipartite begomovirus that spread to India and nearby locales in Southeast Asia during the past two decades. Several studies have investigated potential endogenous host-plant mature microRNAs that may target plant viruses based on in silico criteria [69,70,71,72,73,74,75,76,77]. The miRNAs have evolved as key endogenous biomolecules for regulating gene expression. Several studies have demonstrated the efficacy of amiRNA expression in genetically engineered crops to abate plant virus infection for both RNA and DNA viral genomes [30,31,32,33,37,38,78,79,80]. In this study, mature cassava miRNAs were hybridized in silico with the ICMV-Ker genome to predict the most effective miRNA target binding sites and the specific interactions with the AC1, AC2, AC3, and AV1 of ICMV-Ker.

Three computational algorithms—miRanda, Tapirhybrid, and psRNATarget—predicted a consensus genomic target binding site of mes-miR2950 at locus 639, while all four algorithms identified mes-miR1446a as the most potent cassava miRNA. The MFE is widely used as a standard parameter for miRNA prediction and evolutionary inferences [81]. The MFE of selected miRNA-mRNA target pairs was estimated as −23.11 kcal/mol (miRanda) and −20.30 kcal/mol (RNA22) (Table 3, Table 4 and Table 5), indicating support for miRNA-mRNA duplexes that are predicted to represent ‘true targets’ [82,83], and likely to exhibit robust hybridization (annealing/binding) in the seed region and translation repression [84]. Taken together with free energy measurements, which reflect the dynamic features of miRNAs and their target binding, the combined criteria have identified two predicted cassava mes-miRNAs with robust potential for RNAi and gene silencing of the ICMV-Ker genome when expressed in cassava.

The begomoviral Rep (AC1) is highly conserved protein among geminiviruses. The AC1 gene is essential for viral replication and transcription of ICMV-Ker [85]. In this study, the in silico analyses have implicated the cassava consensus mes-miR1446a in targeting the AC1 encoding region of ICMV-Ker Rep. The cassava precursor of the consensus mes-MiR1446 (Accession ID: MI0024347), located on cassava chromosome Scaffold 12,262 (position 17,213 to 17,343) [86], was identified as an miRNA target mimic in cassava [87], whereas stu-miR1446 has been shown to be targeted by an ABC transporter, resulting in enhanced biotic stress in potato [88].

The cassava-encoded miRNA–ICMV-Ker-mRNA duplex exhibited a low MFE value, which serves as one measure of controlling for false positive results. When combined with results of the ‘four algorithm’ approach to filter false positive targets, the approach was expected to result in a highly sensitive and specific in silico strategy for predicting ‘true’ interactors [89,90,91,92] among the miRNA-mRNA target pairs (Figure 1 and Figure 4) (Table 3).

While interactions between cassava genome locus-derived mes-miRNAs and ICMV-Ker have been established, the ability of cassava mes-miRNAs to control ICMV-Ker is yet to be fully understood. Studies on the interactions between cassava locus-derived mes-miRNAs and ICMV-Ker are vital; they reflect initial steps in the development of miRNA-based anti-plant viral therapies. Future work will focus on the validation of this promising cassava locus-derived mes-miRNA to develop ICMV-Ker-resistance in cassava plants, including evaluating the role of the predicted consensus cassava locus-derived miRNAs in ICMV-Ker replication. The amiRNA construct exhibited high specificity for base-pairing with the target gene, which would be expected to minimize detrimental off-target effects and contribute to stable and reliable protection in subsequent future generations of progeny plants [93]. Further, the small size of amiRNA permits the insertion of a suite of amiRNAs within a single gene expression cassette, enabling the development of transgenic plants, potentially with resistance to multiple viruses [94]. RNAi-screening has emerged as a new approach for elucidating various cellular functions and for predicting host-derived, virus-specific factors [95,96,97]. In this study, 175 experimentally validated cassava miRNAs were evaluated for interactions with annotated targets encoded by the ICMV-Ker genome to establish an effective bioinformatics workflow for predicting ICMV-Ker genome silencing, toward the next steps in developing genetically modified cassava crops with tolerance/resistance that deploys host plant genome encoded miRNAs against the bipartite begomovirus, ICMV-Ker.

## 5. Conclusions and Recommendations

The ICMV-Ker, which infects cassava crops in Southeast Asia, is an emergent begomoviral pathogen associated with an ongoing CMD epidemic that diminishes yield and vigor in all cassava cultivars presently grown in the region. Here, in silico tools and approaches were implemented for predicting effective target binding sites of mature candidate cassava locus-derived mes-miRNAs in the ICMV-Ker genome. Among the 175 cassava miRNAs investigated, predicted hybridization sites were identified within the ICMV-Ker genome. Among them, mes-miR1446a was identified as a highly promising, naturally occurring biomolecule with potential to modulate virus infection and reduce damage to the plant host. Present and future work is focused on the development of ICMV-Ker-resistant cassava plants to abate the effects of CMD. These results demonstrate the utility of this combined in silico-screen-molecular approach to design amiRNA therapies to manage ICMV-Ker in cassava crops and other emergent begomoviral pathogens that threaten food and fiber crops worldwide.

## Figures and Tables

**Table 1:** Summary of algorithms considered for miRNA-mRNA target predictions in this study.

Tools	Algorithms	Organism	Features	Availability
miRanda	Local alignment	Human, rat, fly, worms	Seed pairing, multiple sites and conservation	Web server and source code
RNA22	FASTA	Human, mouse, fly and worms	Pattern recognition and folding energy	Only web server
Tapirhybrid	FASTA	Plants	Seed pairing	Web server and source code
psRNATarget	Smith-Waterman	Plants	Multiple sites, translation inhibition	Only web server
RNAhybrid	Intermolecular hybridization	Any	Seed pairing and free energy	Web server
Targetfinder	FASTA	Plants	Seed pairing	Only source code
Target-align	Smith-Waterman	Plant	Target sites	Web server and source code
TargetScan	Custom made	Mammals, flies, worms, fish	Seed pairing, free energy and conservation	Only Web server
PicTar	FASTA	Vertebrates, flies, worms	Seed pairing, free energy and conservation	Only Web server

**Table 2:** The number of cassava miRNAs to target each gene of ICMV-Ker.

ICMV-Ker Gene	miRanda	RNA22	Tapirhybrid	psRNATarget
AV1	mes-miR2950	mes-miR535(a, b, c, d)	mes-miR160e, mes-miR395e, mes-miR482e, mes-miR535c and mes-miR2950	mes-miR160 (e, f), mes-miR171 (g, h, i, j and k), mes-miR394 (a, b, c), mes-miR397, mes-miR408, mes-miR2111 (a, b) and mes-miR2950
AV2				mes-miR394 (a, b, c)
AC1	mes-miR159 (c, d), mes-miR399e, mes-miR477 (a, b, c, d, e, f, g, h, I and k) and mes-miR1446 (a, b)	mes-miR159 (a, b, c, d), mes-miR319 (a, b, e, f, g, and h), mes-miR395 (a, b, c, d and e), mes-miR1446a and mes-miR2118	mes-miR172 (e, f), mes-miR399e, mes-miR477 (f, g, h), mes-miR482d, mes-miR530 (a, b), and mes-miR1446a	mes-miR159 (a, b), mes-miR395 (a, b, c, d, e), mes-miR397, mes-miR399b, mes-miR530a, mes-miR827, mes-miR1446a and mes-miR2275
AC3	mes-miR390b,	-	mes-miR403 (a, b)	mes-miR403 (a, b)
AC4	mes-miR159 (c, d)	mes-miR159 (a, b, c, d) and mes-miR319 (a, b, e, f, g and h)	mes-miR530 (a, b)	mes-miR159 (a, b), mes-miR397 and mes-miR530a
LIR	mes-miR393 (a, b, c, d)	mes-miR399h	mes-miR395e	-

**Table 3:** Positions of consensus cassava locus-derived mes-miRNAs, predicted by at least three algorithms to target the ICMV-Ker genome.

Cassava miRNA	Position miRanda	Position RNA22	Position TAPIR	Position psRNATarget	MFE * miRanda	MFE ** RNA22	MFE Ratio Tapirhybrid	Expectation psRNATarget
mes-miR159c	2263	2261			−20.75	−19.3		
mes-miR159d	2263	2261			−20.75	−19.3		
mes-miR160e			574	574			0.62	6.5
mes-miR395a		1789		1789		−15.80		6.0
mes-miR395b		1789		1789		−15.80		6.0
mes-miR395c		1789		1789		−15.80		6.0
mes-miR395d		1789		1789		−15.80		6.0
mes-miR399e	2561		2561		−22.90		0.56	
mes-miR403a			1446	1446			0.60	6.5
mes-miR403b			1446	1446			0.60	6.5
mes-miR477f	1592		1592		−23.95		0.54	
mes-miR477g	1592		1592		−23.95		0.54	
mes-miR530a			2425	2425			0.56	6.5
mes-miR1446a	2053	2051	2053	2053	−23.11	−20.30	0.63	6.0
mes-miR2950	639		639	639	−20.15		0.5	6.5

* MFE represnts minimum free energy while MFE ** is the abbreviation of the minimum folding energy.

**Table 4:** Predicted consensus cassava locus-derived mes-miRNA binding sites targeting genes encoded by the ICMV-Ker genome.

miRNA ID	Accession ID	Mature Sequence (5′–3′)	Target Genes ORF(s)	Target Binding Locus Position
mes-miR159c	MIMAT0029177	AUUGGAGUGAAGGGAGCUCUG	AC1/AC4	2261–2281
mes-miR159d	MIMAT0029191	UGGAGAAGCAGGGCACAUGCU	AC1/AC4	2261–2281
mes-miR160e	MIMAT0029183	UGCCUGGCUCCCUGAAUGCCAUC	AV1	574–596
mes-miR395a	MIMAT0029268	CUGAAGUGUUUGGGGGAACUC	AC1	1789–1809
mes-miR395b	MIMAT0029269	CUGAAGUGUUUGGGGGAACUC	AC1	1789–1809
mes-miR395c	MIMAT0029270	CUGAAGUGUUUGGGGGAACUC	AC1	1789–1809
mes-miR395d	MIMAT0029271	CUGAAGUGUUUGGGGGAACUC	AC1	1789–1809
mes-miR399e	MIMAT0029283	UGCCAAAGGAGAUUUGCUCGG	AC1	2581–2581
mes-miR403a	MIMAT0029286	UUAGAUUCACGCACAAACUCG	AC3	1446–1466
mes-miR403b	MIMAT0029287	UUAGAUUCACGCACAAACUCG	AC3	1446–1466
mes-miR477f	MIMAT0029295	AUCUCCCUCAAAGGCUUCCA	AC1	1592–1611
mes-miR477g	MIMAT0029296	AUCUCCCUCAAAGGCUUCCA	AC1	1592–1611
mes-miR530a	MIMAT0029298	UGCAUUUGCACCUGCACCUU	AC1/AC4	2425–2444
mes-miR1446a	MIMAT0029309	UUCUGAACUCUCUCCCUCAU	AC1	2053–2072
mes-miR2950	MIMAT0029305	UUCCAUCUCUUGCACACUGGA	AV1	639–659

**Table 5:** Selected consensus cassava locus-derived mes-miRNA-target pairs predicted by at least three algorithms, with characteristic features of the miRanda and psRNA Target algorithms.

Cassava miRNA	miRNA-Target Pair	Locus Position	MFE (Kcal/mol)	Score	Complementarity (%)	Mode of Inhibition
mes-miR1446a	Query: 3′ uaCUCCCUCUCUCAAGUCUu 5′ |:|||| ||:|||| || Ref:5′ gaGGGGGAAAGGGTTCTGA 3′	2053–2072	−23.11	142	88.24	Cleavage
mes-miR2950	Query: 3’ aggucACACGUUCUCUACCUu 5′ || |||||| ||||| Ref: 5′ catccTGGGCAAGATATGGAt 3′	639–659	−20.15	140	86.67	Cleavage

**Table 6:** The salient parameters of precursor cassava locus-derived mes-miRNAs were determined.

miRNA ID	Length miRNA	Length Precursor	MFE */Kcal/mol	AMFE **	MFEI ***	(G+C)%
mes-MIR1446a	20 nt	131 nt	−52.90	−40.38	−1.01	39.69
mes-MIR2950	21 nt	104 nt	−55.30	−53.17	−1.20	44.23

* MFE is minimum free energy. ** AMFE is the abbreviation of adjusted free energy. *** MFEI is defined as free energy index.

**Table 7:** The free energy (Δ*G*) of the consensus cassava locus-derived mes-miRNA-mRNA target pairs was estimated.

miRNA ID	Accession ID	miRNA-Target Sequence (5′–3′)	ΔG Duplex (Kcal/mol)	ΔG Binding (Kcal/mol)	
mes-miR1446a	MIMAT0029309	5′ UUCUGAACUCUCUCCCUCAU 3′ 5′ GAGGGGGAAAGGGTTCTGAT 3′		−22.80	−21.89
mes-miR2950	MIMAT0029305	5′ UUCCAUCUCUUGCACACUGGA 3′ 5′ CATCCTGGGCAAGATATGGAT 3′		−17.90	−14.82

## Data Availability

Not applicable.

## Supplementary Materials

The following supporting information can be downloaded at: https://www.mdpi.com/article/10.3390/v15020486/s1, Table S1: Cassava mature microRNAs retrieved from miRBase; Table S2: Prediction of effective target binding sites of cassava miRNAs in the ICMV-Ker genome using computational algorithms; Table S3: Gene wise prediction by different algorithms; File S1: Prediction methodology by psRNATarget; File S2: Prediction results of different algorithms in the ICMV-Ker genome and File S3: Construction methodology of Circos plot. Reference [98] was cited in Supplementary Materials.
