# Supplementary material for: In Silico Identification of Cassava Genome-Encoded MicroRNAs with Predicted Potential for Targeting the ICMV-Kerala Begomoviral Pathogen of Cassava
Source: Viruses. 2023 Feb 9;15(2):486. doi: 10.3390/v15020486 (PMC9963618; doi:10.3390/v15020486)
Supplement: Supplementary file 1 [file viruses-15-00486-s001.zip › Circos Plot-File S3.pdf]

Circos Plot

The inter-relationship between cassava locus-derived mes-miRNAs and ICMV-Ker ORFs as visualized by chord diagrams using the "circlize" package. Biological data were analyzed to generate chord diagram (Circos plot) using R software version 4.2.0 (Vienna, Australia) [1]. It supports using the grammar of graphics syntax to present data as follows:

Library (circlize)

chordDiagram (data, grid.col = ”white”,

annotationTrack= "grid')

Reference

1. R Core Team. R: A Language and Environment for Statistical Computing; R Foundation for Statistical Computing: Vienna, Austria, 2022. Available online: https://www.R-project.org/ (accessed on 30 May 2022).
