# Supplementary material for: In Silico Identification of Cassava Genome-Encoded MicroRNAs with Predicted Potential for Targeting the ICMV-Kerala Begomoviral Pathogen of Cassava
Source: Viruses. 2023 Feb 9;15(2):486. doi: 10.3390/v15020486 (PMC9963618; doi:10.3390/v15020486)
Supplement: Supplementary file 1 [file viruses-15-00486-s001.zip › psRNATarget-File S2.pdf]

psRNATarget

psRNATarget is a high-performance plant small RNA target analysis server and can be accessed at (https://www.zhaolab.org/psRNATarget/analysis?function=2). It enables transmitting and analyzing 'big' data. The server is based on new scoring schema that is capable of discovering miRNA-mRNA interactions at higher 'recall rates'. The psRNATarget V2 has clear, compelling and user-friendly interfaces that enhance user experiences and present data clearly and concisely. The psRNATarget server is utilizing selected published plant miRNAs based on miRBase 'Release 21, June 2014'. In the current study, 153 experimentally validated published cassava (*Manihot esculenta*) locus-derived mes-miRNAs were employed for analysis.

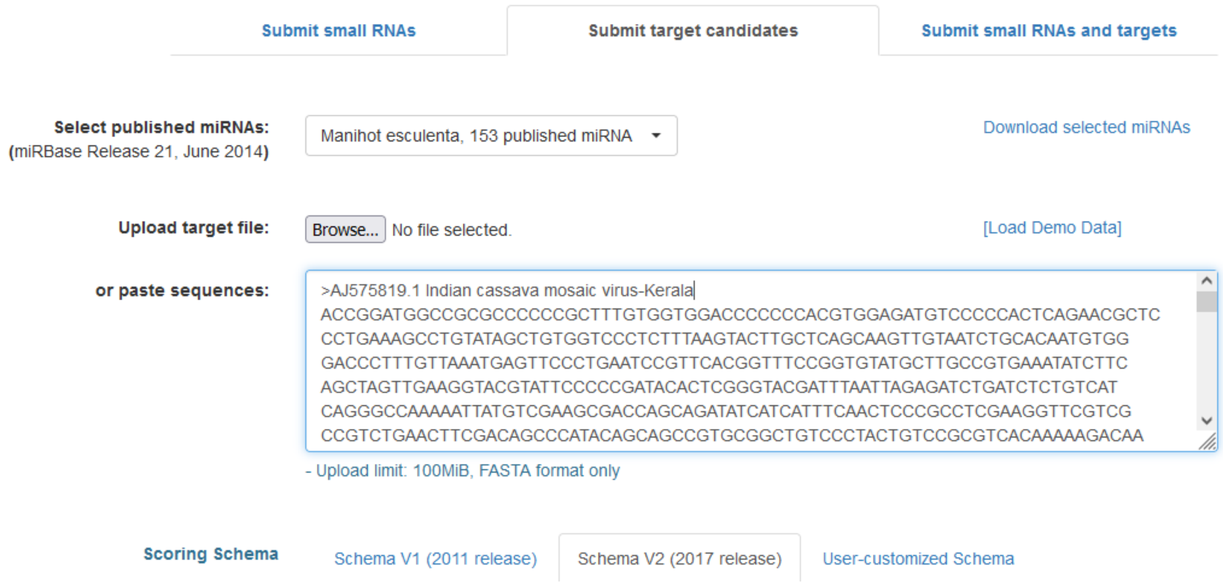

Scoring Schema — Schema V1 (2011 release) | Schema V2 (2017 release) | User-customized Schema

**# of top targets:**	200	**Expectation:**	7
**Penalty for G:U pair:**	0.5	**Penalty for other mismatches:**	1
**Extra weight in seed region:**	1.5	**Seed region:**	2 - 13 NT
**# of mismatches allowed in seed region:**	2	**HSP size:**	19

☑ Allow bulge(gap)

**Penalty for opening gap:**	2	**Penalty for extending gap:**	0.5

☐ Calculate target accessibility

~~**Max UPE:**~~	~~25~~	~~**Flank length:**~~	~~17 / 13 NT in up/downstream~~

Translation inhibition range: 10 NT - 11 NT

Upload & Submit
